# Diabetes Mellitus Prediction and Severity Level Estimation Using OWDANN Algorithm

**DOI:** 10.1155/2021/5573179

**Published:** 2021-08-20

**Authors:** Annamalai R, Nedunchelian R

**Affiliations:** ^1^Department of Information Technology, Jeppiaar Institute of Technology, Kanchipuram 631604, India; ^2^Department of Computer Science and Engineering, Excel Engineering College, Namakkal 637303, India

## Abstract

Today, diabetes is one of the most prevalent, chronic, and deadly diseases in the world owing to some complications. If accurate early diagnosis is feasible, the risk factor and incidence of diabetes may be greatly decreased. Diabetes prediction is stable and reliable, since there are only minimal labelling evidence and outliers found in the datasets of diabetes. Numerous works coped with diabetes disease prediction and provided the solution. But the existing methods proffered low accuracy detection and consumed more training time. So, this paper proposed an OWDANN algorithm for diabetes mellitus disease prediction and severity level estimation. The proposed system mainly consists of two phases, namely, disease prediction and severity level estimation phase. In the disease prediction phase, the preprocessing is performed for the Pima dataset. Then, the features are extracted from the preprocessed data, and finally, the classification step is performed by using OWDANN. In the severity level estimation phase, the diabetes positive dataset is preprocessed first. Then, the features are extracted, and lastly, the severity level is predicted using GDHC. The extensive experimental results showed that the proposed system outperforms with 98.97% accuracy, 94.98% sensitivity, 95.62% specificity, 97.02% precision, 93.84% recall, 9404% *f*-measure, 0.094% FDR, and 0.023% FPR compared with the state-of-the-art methods.

## 1. Introduction

Nowadays, a large volume of data is being generated from healthcare industries. These data may be organised or unstructured data. Various documents are kept in hospitals, such as details from patients' profiles, X-ray images, and reports from numerous diagnostic examinations [1]. Today, diabetes mellitus (DM) [2] is a big global and rising problem [3] in developing countries such as India. Diabetes mellitus of the circulatory system is chronic, persistent ailment triggered by excessive sugar levels. It can be classified into four types [4–6]. Type 1 diabetes is known as juvenile diabetes or insulin-dependent diabetes which occurs when the immune system of the body damages the insulin release cell; finally, it removes insulin production from the body [7]. In type 2 diabetes, insulin will not be produced in an adequate amount, which will not be sufficient for body needs, which leads to type 2 diabetes. For the most part, it happens at 40 years of age [8]. Next, gestational diabetes mellitus (GDM) [9] is associated with adverse perinatal outcomes [10]. The last one is pregestational diabetes [11] that is diagnosed before pregnancy. Patients ought to measure their blood glucose and rely on their wellbeing for protective precautions at least two times a month [12].

It has an effect on the numerous sections of the organism which involves pancreas glitch, danger of cardiac attacks, high blood pressure, kidney deceit, pancreatic problems, nervous damage, foot problems, ketoacidosis, vision disturbance and other eye problems, waterfalls, and glaucoma [13]. It must also be tested and it is incurable. An individual who is diabetic can experience serious complications such as nerve injury, heart attack, kidney failure, or stroke [14]. So, disease prediction is very important. Also, the early identification of individual risk for diabetes is essential for effective targeting of preventive measures [15]. Most of the patients describe their symptoms using superlative degree, such as “never, very, rarely, often, sometimes, always, and most of the times” and each of the specific symptoms can be graded using linguistic variables like “mild,” “moderate,” “high,” and “low” [16]. The need to classify the high-risk citizens of the remainder of the society correctly and economically must therefore be concentrated on [17].

In recent years, many academics have proposed a method for disease prediction utilising machine learning algorithms. Some machine learning algorithms [18] are Linear Discriminant Analysis (LDA), Quadratic Discriminant Analysis (QDA), Naive Bayes (NB), Support Vector Machine (SVM) [19], Gaussian Process Classification (GPC), Artificial Neural Network (ANN) [20], AdaBoost (AB), Logistic Regression (LR), Decision Tree (DT), and Random Forest (RF) with different dimensionality reduction and cross-validation techniques [21]. But it has more training time and provides less accuracy than the deep learning algorithms. The abovementioned problems are taken and to trounce these issues, this paper proposed an efficient deep learning algorithm to predict the diabetes mellitus and severity level estimation.

The remainder of the paper is organised as follows: Section 2 discusses the work that is related to diabetes mellitus and severity level analysis.  Section 3 provides clear descriptions of the proposed system. It states diabetes disease prediction and severity level estimation.  Section 4 explains the results that are obtained from the used approaches and model evaluation and compares them with existing methodologies, and Section 5 concludes the work and future direction of the proposed system.

## 2. Related Work

Li et al. [22] have incorporated CRNNs for the prediction of glucose. The model was trained mainly on data that included Continuous Glucose Meter (CGM), carbohydrate, and insulin. Following the preprocessing of the blood glucose (BG), carbohydrate, and insulin (other considerations may also be considered) time allocations, CRNN were supplied for preparation. Lastly, the machine is diabetic or otherwise graded. There were four algorithms that indicate the efficiency of the repeating coevolutionary neural network, as well as better output than current methods. However, the efficiency of hypoglycaemia prediction has declined even more rapidly than that of hyperglycaemia prediction as the horizon of forecast rises.

Pei et al. [23] recommended a precised and rapid diabetes mellitus screening model. In order to classify patients with diagnosis based on nine noninvasive and easily obtained clinical features like race, age, gender, Body Mass Index (BMI), and hypertension, the history of cardiovascular disease or stroke, family history of diabetes, physical activity, and job stress and salt stress, the method has used five common classifications (J48, AdaBoostM1, Sequence Minimal Optimization). The findings revealed that J48 decision tree classification had the highest value relative to other classifiers and can be used without the need for intrusive testing to check people for the possibility of early diabetes. However, it was challenging to evaluate the performance from just one major hospital in China.

Martinsson et al. [24] proposed a blood glucose forecast with variation estimate focused on recurrent neural networks. The solution was focused on repeating, eventually qualified neural networks, which only required the background of the patient with glucose level. Results were contrasted with the existing state of the art for the estimation of blood glucose level in the Ohio T1DM dataset. The device provided an estimation of its certainty as well as the predicted glucose benefit, allowing users to perceive the forecast levels. The training of the recurring neural network to parametrize the Gaussian univariate distribution over the output was carried out. The solution did not involve functionality engineering or preprocessing of data and was computer-cheap. However, the machine was practising longer.

The profound physiological model for T1DM blood glucose prediction was given by Munoz-Organero et al. [25]. The carbohydrate differential equations and the physiologic model for insulin absorption were designed based on the long term memory (LSTM) cell-specific recurring neural network (RNN). The findings revealed that the RMSE values for hypothetical patients were below 5 mg/dL and that, for actual patients, the RMSE values were below 10 mg/dL. In comparison to state-of-the-art approaches, the device has done higher. The dataset had, nonetheless, the amount of days for each participant to report the results. It was also impossible for the model to estimate potential BG values.

Mahabub [26] proposes a rigorous diabetes prediction voting strategy focused on conventional approaches. Here, eleven popular machine learning algorithms such as the Naive Bayes, the KNN, Support Vector Machine, Random Forest, Logistic Regression, Gradient Boosting, Ada Boosting, and several more have been used for early detection of diabetes. The 11 algorithms were analysed using different criteria, such as performance, precision, *f*-measurement, and reminder. After cross-validation and hypertuning, the best three machine learning algorithms were designed and employed in the Ensemble Voting Classifiers. The experimental results confirmed the Pima Indians Diabetes Database's good results, which are around 86 percent correct. The precision was comparatively good, but the estimation outcomes were not 100 percent, and the preparation period was high.

Zhou et al. [27] studied the diabetes prediction using an initial deep neural network. First, data has been split into preparation and evaluation data. Next, people were categorised depending on their medical problems. The tests proved the efficacy and efficiency of the method. The highest accuracy data collection for diabetes was 94.02174%, and the most reliable was that for Pima Indians at 99.4112%. A number of studies have been performed with Pima Indians diabetics. The experimental findings demonstrated major advances in the existing state-of-the-art equipment. The machine can only manage tiny volumes of info.

Postorino and Versaci [28] introduced the fuzzy cluster strategy, as an alternate method to previous crisp approaches, is based on the potentiality it can give when two characteristics of fuzziness are examined. The first involves identifying an airport as a fuzzy set, which can subsequently be described using not only numerical values but also linguistic characteristics. The second point examined is distance, which is somewhat ambiguous quantity. However, because different criteria can result in drastically different clusters, some of the most important criteria are briefly outlined.

Mahmoudi et al. [29] presented a number of regression models with fractional Brownian motion errors that can be used to cluster data. The main goal is to group these models and then find a subset of affordable predictors that can reasonably predict a response variable. In addition, the fuzzy clustering method was used to group the models under consideration.

## 3. Diabetes Mellitus Disease Prediction and Severity Level Estimation Using Deep Artificial Neural Network

The incurable metabolic condition, diabetes mellitus or diabetes, is attributed to loss or absence of an insulin hormone. The cells will consume the glucose (blood sugar) from food sources and have the requisite amount of energy. This is an important hormone generated by the pancreas. If unchecked, diabetes can become lethal, and it invites several other diseases explicitly or indirectly, including cardiac attack, heart failure, stroke, and more. Therefore, diabetes diagnosis and severity evaluations are very necessary to avoid more problems and take timely precautions and the development of the condition. Healthcare firms collect a vast volume of data, including electronic databases, photographs, weak data, and text, but it remains a significant challenge to obtain information and insight into the data. This paper proposed an efficient deep learning approach for diabetic disease prediction and severity level estimation. The proposed system collects the data to predict diabetic patients from the Pima Indians datasets.

**Figure 1:**
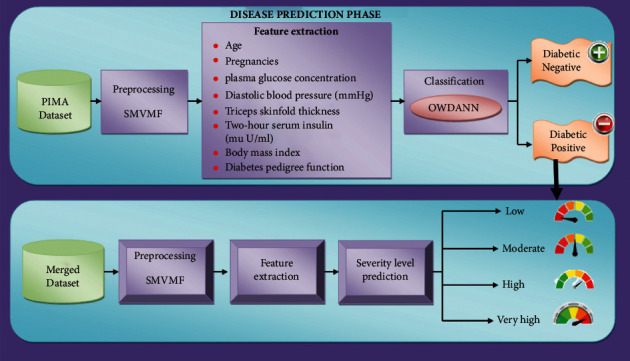
Architecture diagram for the proposed methodology.

The proposed system consists of two phases, namely, the disease prediction phase and severity level estimation phase. In the disease prediction phase, the proposed system collects data from the Pima Indians datasets to anticipate diabetic patients, which is then preprocessed using the switching midvalue-based morphological Filter (SMVMF), followed by a feature extraction stage that summarizes the majority of the information present in the original feature set. After identifying the critical features, the classification step is performed, which efficiently classifies the patient results into positive and negative classes by utilising the OWDANN algorithm to determine if the patient possesses diabetes or not. In the severity level estimation phase, the illness data is blended with common attributes such as sex, age, and other factors to generate a new merged dataset, which is then preprocessed and feature extracted again. Finally, using the GDHC algorithm, the severity level is predicted and categorised as “low,” “moderate,” “high,” and “very high.” These phases are elaborately explained in the following sections. Block diagram of the proposed method is outlined in Figure 1.

### 3.1. Disease Prediction Phase

The disease prediction phase is the initial phase to predict the disease as positive or negative. The disease prediction phase consists of four phases, say, data collection, preprocessing, feature extraction, feature selection, and classification. These phases are explained in the following sections.

#### 3.1.1. Data Collection

The proposed system collects the data from the Pima Indians dataset which is taken from the UCI Repository.

There are also numeric properties in the dataset. Mathematically, it is expressed as follows:

(1) $$\begin{array}{c} {\tilde{A}}_{ds}^{\prime \prime }=\left\{{\tilde{a}}_{1}^{\prime \prime },{\tilde{a}}_{2}^{\prime \prime },{\tilde{a}}_{3}^{\prime \prime },..........{\tilde{a}}_{k}^{\prime \prime }\right\}, \end{array}$$

where ${\tilde{A}}_{ds}^{\prime \prime }$ indicates the diabetic patient dataset and ${\tilde{a}}_{k}^{\prime \prime }$ signifies *k* number of attributes in the Pima Indians dataset. It is used for further processing.

#### 3.1.2. Preprocessing

Preprocessing is an important step to reduce the noise in the signal and smooth the data values. Hither, preprocessing is performed by using the switching midvalue-based morphological Filter (SMVMF). Switching midvalue filter is used to effectively remove the noise and restore the corrupted data efficiently. The time gap between the forecast glucose and the recorded glucose levels often decreases. If the noise and their position are detected in the dataset, then it is easy to replace the noisy value with the best estimate of the good value. It gives better performance in comparison with the median filter. But the existent switching midvalue filter is streaking at higher noise densities, and also, it could not smooth the signal values. To smooth the signal values and provide less noise density, the proposed system used a morphological operation to smooth the signal concurrently by suppressing all positive and negative signal results. The algorithmic procedures of the SMVMF algorithm are described as follows:

Step 1: some attributes in the dataset are based on the glucose concentration signal values. So, it initially takes the collected data from the dataset (${\tilde{A}}_{ds}^{\prime \prime }$) and then determines the initial window size ${\tilde{B}}_{wv}$ using the following equation:
  (2) $$\begin{array}{c} {\tilde{B}}_{wv}=\left\{\begin{array}{c} {\tilde{C}}_{nd}\leq 40,\\ 41>{\tilde{C}}_{nd}<60,\\ {\tilde{C}}_{nd}\geq 61, \end{array}\right) \end{array}$$
where ${\tilde{C}}_{nd}$ denotes the noise density.
 Step 2: this step starts by dividing both the noisy values (*d*_noisey_) and the corresponding binary values (*d*_binary_) from ${\tilde{A}}_{ds}^{\prime \prime }$into $\left(\left(2{\tilde{B}}_{wv}+1\right)\times \left(2{\tilde{B}}_{wv}+1\right)\right)$ sliding windows.
 Step 3: then, calculate the total number of signal values (${\tilde{E}}_{\text{tot}}$) which is based on the number of noisy signal values (${\tilde{E}}_{ns}$) and the number of noisy free signal values (${\tilde{E}}_{nfs}$) in the current filtering window of *d*_noisey_using *d*_binary_. Mathematically, it is represented as follows:
  (3) $$\begin{array}{c} {\tilde{E}}_{\text{tot}}={\tilde{E}}_{ns}+{\tilde{E}}_{nfs}. \end{array}$$
 Step 4: next, replace the noisy signal values with the median of noiseless signal values. It is expressed as follows:
  (4) $$\begin{array}{c} {\tilde{E}}_{\text{noiseless}}^{s}\leftarrow {\tilde{E}}_{\text{noisy}}^{s}. \end{array}$$
 Step 5: finally, to smooth the signal, the morphological operation is applied. The good and detrimental effects on the signal are completely removed. Dilution and erosion are two specific types of mathematical morphological (MM) operations that can be referred to as symbols ⊕ and Θ. Here, erosion of ${\overset{↔}{F}}_{mor}$ by ${\tilde{E}}_{\text{noiseless}}^{s}$ is the set of all points *z* such that ${\tilde{E}}_{\text{noiseless}}^{s}$, translated by *z*, is contained in ${\overset{↔}{F}}_{\text{erosion}}$.
  (5) $$\begin{array}{c} {\overset{↔}{F}}_{\text{mor}}\Theta {\tilde{E}}_{\text{noiseless}}^{s}=\left\{z|\left({\tilde{E}}_{\text{noiseless}}^{s}\right)z\subseteq {\overset{↔}{F}}_{\text{mor}}\right\}. \end{array}$$

Dilation of ${\overset{↔}{F}}_{mor}$ by ${\tilde{E}}_{\text{noiseless}}^{s}$ in *z* is denoted by ${\overset{↔}{F}}_{\text{mor}}⊕{\tilde{E}}_{\text{noiseless}}^{s}$. Dilation of ${\overset{↔}{F}}_{mor}$ is done by the structuring element ${\tilde{E}}_{\text{noiseless}}^{s}$ in the set of all displacements *z*, such that $\left(\overset{⟶}{{\tilde{E}}_{\text{noiseless}}^{s}}\right)$ and ${\overset{↔}{F}}_{\text{mor}}$ overlap by at least one element.

(6) $$\begin{array}{c} {\overset{t}{F}}_{\text{mor}}⊕{\tilde{E}}_{\text{noiseless}}^{s}=\left\{z|{\left(\overset{⟶}{{\tilde{E}}_{\text{noiseless}}^{s}}\right)}_{z}\cap {\overset{t}{F}}_{\text{mor}}\neq \varphi \right\}. \end{array}$$

These operators are used to smooth the noiseless signal values. The accessible closing operator and the closing operator are represented on the basis of the sequential opening and closing operation as

(7) $$\begin{array}{c} {\overset{t}{F}}_{\text{mor}}\circ {\tilde{E}}_{\text{noiseless}}^{s}=\left({\overset{t}{F}}_{mor}\Theta {\tilde{E}}_{\text{noiseless}}^{s}\right)⊕{\tilde{E}}_{\text{noiseless}}^{s},\\ {\overset{t}{F}}_{\text{mor}}\cdot {\tilde{E}}_{\text{noiseless}}^{s}=\left({\overset{t}{F}}_{mor}⊕{\tilde{E}}_{\text{noiseless}}^{s}\right)\Theta {\tilde{E}}_{\text{noiseless}}^{s}. \end{array}$$

By using the above equations, the signal is smoothed and cleared for further processing. In addition, if any records include unrecorded values, they will be filled by replacing the missing value for a given attribute with the average value, i.e. the median value for that attribute. Mathematically, it is expressed as follows:

(8) $$\begin{array}{c} G_{mv}^{\prime \prime }=\frac{\sum {\tilde{A}}_{ds}^{\prime \prime }}{n}, \end{array}$$

where *G*_*mv*_^″^ indicates the missing attributes, ${\tilde{A}}_{ds}^{\prime \prime }$ signifies the collected dataset, and *n* represents the number of data pieces in the dataset.

#### 3.1.3. Feature Extraction

The feature extraction method is carried out at this phase. The extraction of characteristics improves the precision of the models trained by extracting features from the results. The general structure process decreases the dimension of the data by eliminating redundant data. In the proposed diabetes mellitus prediction system, significant variables such as pregnancy age (years), plasma glucose concentration, diastolic blood pressure (mm Hg), triceps skinfold thickness, two-hour serum insulin (mu U/ml), body mass index, and diabetes pedigree function are captured from the dataset. The extracted features are represented as

(9) $$\begin{array}{c} {\left(H_{fset}^{\prime \prime }\right)}_{z}=\left\{h_{1}^{\prime \prime },h_{2}^{\prime \prime },h_{3}^{\prime \prime },...................h_{s}^{\prime \prime }\right\}, \end{array}$$

where *H*_*fset*_^″^ indicates the feature set that has been extracted and *h*_*s*_^″^ signifies the *s* numeral of features that are extracted from the preprocessed dataset.

#### 3.1.4. Classification Phase

**Figure 2:**
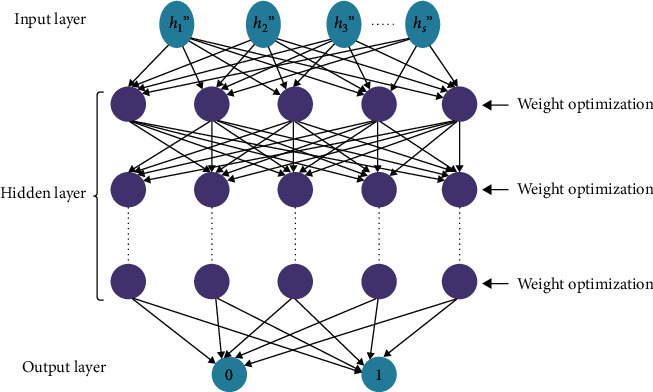
Structure diagram for the OWDANN algorithm.

After extracting features from the preprocessed dataset, the classification process is done. By determining whether the patient has diabetes or not, it efficiently categorises the patient's data into positive and negative categories. In this case, a positive class means a diabetic patient is the source of the specific results, while the negative class implies a nondiabetes patient is the output. The values should be 1 and 0, with 1 being a positive result and 0 being a negative result. The classification is done by using the optimal weighted based deep artificial neural network (OWDANN) algorithm. Normally, the artificial neural network (ANN) is a pretty famous algorithm and the weighted guided graphs may be better understood, where nodes are generated by artificial neurons and the relation of neuron outputs and neuron inputs by directed borders of weight may be described. To increase the hidden layer in ANN, it employs a deep learning concept. The Deep Artificial Neural Network (DANN) provided satisfactory results, but it selects the weight values randomly. To select the optimal weights for the corresponding data, the Blue Monkey Optimization (BMO) algorithm is used. So, the proposed classification algorithm is termed as the OWDANN algorithm. Also, to increase the accuracy of the proposed system, the activation function is utilised by tan*h* function. The structural diagram for the OWDANN algorithm is shown in Figure 2.

Figure 2 outlines the three layers in the OWDANN algorithm: the input layer, the hidden layer, and the output layer. The input layer takes the data input and extracts the values and transfers them to the cached layer. Hidden layers allow the neural network feature to be separated into separate data transformations. Each feature in the hidden layer is specialised in a given output. Since this method has many layers and a wide number of inner nodes, it is costly computationally but promises encouraging results after model training. The algorithmic procedures of the OWDANN algorithm are explained as follows.

Initially, the DANN algorithm takes the extracted feature set as input with their equivalent random weight value and sums all the multiplied values. Weights state the strength of the connection between neurons and decide how much influence the given input will have on the neuron's output. If the weight *j*_1_^″^ has a higher value than the weight *j*_2_^″^, then the input *h*_1_^″^ will have a higher influence on the output than *j*_2_^″^ [30].

(10) $$\begin{array}{c} {\hat{I}}_{z}^{\prime \prime }=\left(h_{1}^{\prime \prime }\times j_{1}^{\prime \prime }\right)+\left(h_{2}^{\prime \prime }\times j_{2}^{\prime \prime }\right)+........+\left(h_{s}^{\prime \prime }\times j_{s}^{\prime \prime }\right). \end{array}$$

The above equation is rewritten in dot product as follows:

(11) $$\begin{array}{c} {\hat{I}}_{z}^{\prime \prime }={\left(H_{fset}^{\prime \prime }\right)}_{z}.{\left(J_{wv}^{\prime \prime }\right)}_{z}, \end{array}$$

where (*H*_*fset*_^″^)_*z*_ indicates the extracted feature set and (*J*_*wv*_^″^)_*z*_ signifies the corresponding random weight values for the inputs (i.e., extracted feature set). These random weight values are not efficient for accurate disease prediction. To provide the optimal weight value, the system uses a Blue Monkey Optimization (BMO) algorithm. In the first epoch, the random weights (*J*_*wv*_^″^)_*z*_ and monkey power rates (*J*_rate_)are initialized with BMO and also initialize the blue monkey and the children population as [31]

(12) $$\begin{array}{c} K_{bm}^{\prime \prime }=\left\{k_{1}^{\prime \prime },k_{2}^{\prime \prime },k_{3}^{\prime \prime },..................k_{n}^{\prime \prime }\right\},\\ K_{bc}^{\prime \prime }=\left\{k_{1}^{\prime \prime },k_{2}^{\prime \prime },k_{3}^{\prime \prime },..................k_{n}^{\prime \prime }\right\}, \end{array}$$

where *K*_*bm*_^″^ indicates the blue monkey population and represents their children. Choose the worst value and the best fitness benefit for each category and hold it at the current best, and choose the best health for children. Then change the position of each blue monkey in the group, which is defined by the best blue monkey position, by the following equations:

(13) $$\begin{array}{c} {\left(J_{\text{rate}}\right)}_{m+1}=\left(\alpha *{\left(J_{\text{rate}}\right)}_{m}\right)+\left(L_{w}^{\prime \prime }-J_{wv}^{\prime \prime }\right)*Rand*\left(L_{\text{best}}^{\prime \prime }-L_{m}^{\prime \prime }\right),\\ L_{m+1}^{\prime \prime }=L_{m}^{\prime \prime }+{\left(J_{\text{rate}}\right)}_{m+1}*Rand, \end{array}$$

where *α* signifies the constant value, *L*_*w*_^″^ indicates the leader weight, *J*_*wv*_^″^ denotes the random weight value for the extracted features set, *L*_*m*_^″^ signifies the monkey position, *L*_best_^″^ represents the leader position, and *Ran*  *d* indicates an arbitrary number between [0, 1]. In order to update the children of blue monkey, the following equations are used:

(14) $$\begin{array}{c} {\left(J_{\text{rate}}^{b}\right)}_{m+1}=\left(\alpha *{\left(J_{\text{rate}}^{b}\right)}_{m}\right)+\left({\left(L_{w}^{\prime \prime }\right)}^{b}-J_{wv}^{\prime \prime }\right)*Ran\;d*\left({\left(L_{\text{best}}^{\prime \prime }\right)}^{b}-{\left(L_{m}^{\prime \prime }\right)}^{b}\right), \end{array}$$

where *J*_rate_^*b*^ signifies the child power rate, (*L*_*w*_^″^)^*b*^ indicates the leader child weight, (*L*_best_^″^)^*b*^ represents the leader child position, and (*L*_*m*_^″^)^*b*^ represents the child position. The position should be updated in each iteration. Finally, evaluate the best fitness of each blue monkey and children, which is the optimal weight value for the extracted feature set. Based on this fitness level, the system selects the optimal weight value for each feature in the extracted feature set. It is expressed as follows:

(15) $$\begin{array}{c} {\left(M_{owe}^{\prime \prime }\right)}_{z}=\frac{1}{1+{\left(J_{\text{rate}}\right)}_{m}+{\left(J_{\text{rate}}^{b}\right)}_{m+1}}, \end{array}$$

where (*M*_*owe*_^″^)_*z*_ represents the optimal weight value for the extracted feature set. If the new best is better than the current best value, then update the new optimal value. After that, these optimal weight values and the extracted feature set are given to the first hidden layer. It is represented mathematically as

(16) $$\begin{array}{c} {\left(H_{lay}^{1}\right)}_{z}=\sum_{z=1}^{n}{\left(H_{fset}^{\prime \prime }\right)}_{z}.{\left(M_{owe}^{\prime \prime }\right)}_{z}. \end{array}$$

Then activation function is calculated. It is employed by tan*h* activation function to increase the accuracy level. With tan*h* it is possible to balance negative feedback strongly and to map zero input close to zero. It is described as follows:

(17) $$\begin{array}{c} {\left(N_{af}^{\prime \prime }\right)}_{z}=f\left(\sum_{z=1}^{n}{\left(H_{fset}^{\prime \prime }\right)}_{z}.{\left(M_{owe}^{\prime \prime }\right)}_{z}.{\left(O_{f}\right)}_{z}\right),\\ {\left(O_{f}\right)}_{z}=\tan\;h\left({\left(H_{fset}^{\prime \prime }\right)}_{z}\right). \end{array}$$

After that, compute the output by summing up all the input values with their optimal weight value and the bias values.

(18) $$\begin{array}{c} {\left(P_{ol}^{\prime \prime }\right)}_{z}=Bias+\sum_{z=1}^{n}{\left(H_{lay}^{1}\right)}_{z}.{\left(M_{owe}^{\prime \prime }\right)}_{z}. \end{array}$$

Finally, the loss function is calculated, which squares the difference between the actual predicted value (*P*_*ol*_^″^)_*z*_ and the targeted output (*a*_*pv*_)_*z*_. It is expressed as follows:

(19) $$\begin{array}{c} \text{Loss}=\left\|{\left(a_{pv}\right)}_{z}-{\left(P_{ol}^{\prime \prime }\right)}_{z}\right\|. \end{array}$$

**Figure 3:**
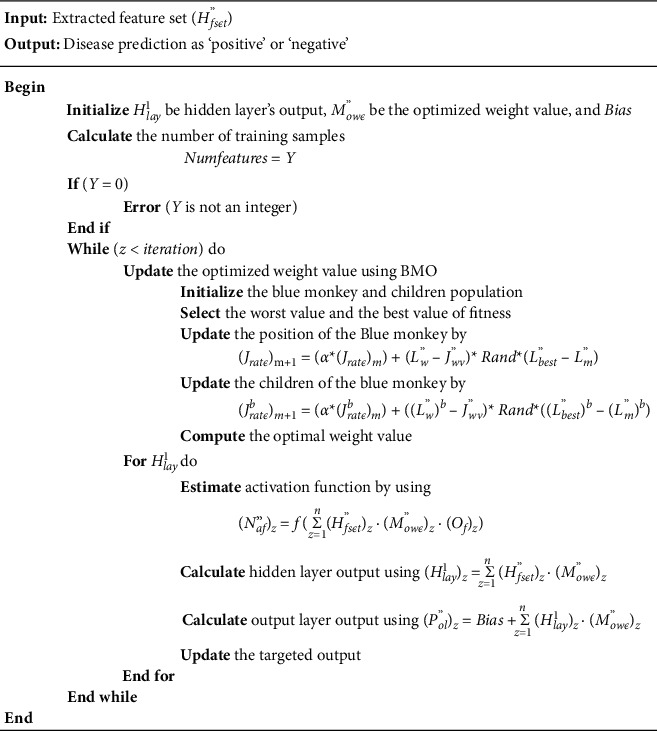
Pseudocode for the optimal weighted based deep artificial neural network (OWDANN) algorithm.

Here, the minimum value is set as a threshold for the loss function. If the initialized threshold value met this fitness, then the output is expressed as the final output; contrarily, the position of the weight value is renewed and the best weight value is chosen by employing the same BMO. Again, the output unit is determined based on this OWDANN algorithm, and the output data is trained for the classification process. The pseudocode for the OWDANN algorithm is elucidated in Figure 3.

### 3.2. Severity Level Estimation Phase

After predicting the disease, estimation of the risk level is very important to control diabetes mellitus. Diabetes may cause several problems, especially in the head, eyes, heart, kidneys, and nerves that may influence certain areas of the body. As a result, determining the severity of a patient's prognosis plays a critical role in diabetes management and early detection. This phase is used to ascertain the severity level estimation after 3 years (age). This phase comprises the following stages: preprocessing, feature extraction, and severity level prediction.

#### 3.2.1. Diabetic Patient Dataset

To build a new dataset, the patient with disease data is combined with some common features such as sex, age, and so on. This new dataset is regarded for simulation.

#### 3.2.2. Preprocessing and Feature Extraction

The preprocessing step included in the above disease prediction phase is done for this new dataset. Following that, significant features such as blood pressure (BP), optic disc diameter, anaemia, numbness, blood urea, and blood sugar are recovered from the new diabetic dataset for effective analysis in feature extraction. The selected feature set is signified by the notation (*Q*_new_^″^)_*x*_.

#### 3.2.3. Severity Level Prediction

The retrieved characteristics are used as input in this phase to the Great-circle Distance-based Hierarchical Clustering (GDHC) algorithm to predict the severity level. It groups severity level as “low,” “moderate,” “high,” and “very high.” The GDHC clustering algorithm groups the severity level based on the score values. These values are tabulated in Table 1.

Using Table 1 details, the GDHC algorithm groups the severity level (low or moderate or high or very high). Clustering Hierarchical (HC) is an algorithm that groups identical artefacts into clusters. The endpoint is a community of clusters in which each cluster is different, and the artefacts inside each cluster are quite close. But it rarely provides the best solution and normally, the existing HC algorithm uses Euclidean distance to calculate the distance between cluster points, but it did not provide effective results. So the system uses great-circle distance formula to provide better and accurate results. The algorithmic procedures of the GDHC algorithm are described in the following steps:

Step 1: decide the number of clusters (*R*_*c*_^″^) from the extracted feature set (*Q*_*new*_^″^)_*x*_.
Step 2: next, select *Sr*_*po*_ points from the cluster as centroids
* *Step 3: assign all the points to the nearest cluster centroids (*T*_*nc*_^″^).
* *Step 4: after that, compute the centroids of newly formed clusters (i.e., calculate the distance between each data point and cluster centres). Hither, the distance will be calculated by using the great-circle distance formula. It is represented as
  (20) $$\begin{array}{c} U_{\text{dist}}=\sqrt{hav\left(T_{nc}^{\prime \prime }-Sr_{po}\right)}, \end{array}$$
* *where *U*_dist_ signifies the distance between each data point and cluster centres.
* *Step 5: repeat steps 3 and 4.

#### 3.2.4. Statistical Testing

By assigning the items in the dataset to *k* fuzzy classes, fuzzy classification can minimize the dimensionality of multivariate datasets [32–35]. As a result of this procedure, a new dataset is created in which the original spatial coordinates are defined just by membership in the *k* classes. According to the Central Limit Theorem, any finite variance distribution with a larger sample size (*n* > 50) will have a sample mean that is roughly normal. And the natural distribution of the samples is assumed in our analysis and estimates, observing and contrasting the binary value distribution with the opinion distribution (positive, negative, and neutral) (only positive and negative). Using the *Z* test (percentage) for binary significance (only positive, negative, and neutral) data from the dataset to test the following hypothesis: The null hypothesis asserts that positive and negative integers are the same. *P* = 0.5 indicates that the value = 1 or “PASS”. H1 (alternative theory): Positive and negative numbers are not the same thing. *P* = 0.5 denotes a value of 0 or “FAIL.” Simulating the rest of the data is done in the same way. In the chi-squared test, these simulated findings are employed. The following is the hypothesis: is there a null hypothesis? H0: opinions are plentiful. The terms “observed” and “expected” are interchangeable. H1 (alternative hypothesis): opinions are unfavourable (biased). The terms “observed” and “expected” are not interchangeable. The actual data is the observed opinion, while the expected values are the randomly created simulated data. In total, 22 out of 100 samples fail the test. In the case of neutral excluded (Blue-bottom), 22 out of 100 people failed the test. As a result, we can conclude that the chi-squared test was passed by the majority of the sample. As a result, we accept opinion/sentiment as the null hypothesis. After that, an Analysis of Variance (ANOVA) test was used to see if there was any difference in the data. The following is the hypothesis: H0 (null hypothesis): Opinions do not differ considerably. H1 (alternative hypothesis): Opinions differ greatly. All samples were subjected to the test. Since *F* cal = 51.463 > *F* tab (0.05) (24.126) = 1.463, the result is as follows: as a result, H0 is denied, whereas H1 is approved.

## 4. Results and Discussion

In this section, the performance of the proposed efficient deep learning approach for diabetes mellitus disease prediction and severity level prediction system is analysed. The system is implemented in the working platform of MATLAB. The dataset descriptions are described in Section 4.1. Sections 4.2 and 4.3 explain the results of classification (OWDANN) and clustering (GDHC), respectively. Here, the results are compared with some existing methodologies.

### 4.1. Dataset Description

Pima Indians women's diabetes dataset was included in the proposed framework. The proposed study is a reasonable dataset to test because only a few datasets exist in this region. More related signs linked with diabetes are found in the data collection. Since 1965 the national institute of diabetes and digestive and kidney disorder has developed this dataset under an extended analysis. The dataset often consists of 8 numerically measured attributes (pregnancies, cholesterol, blood pressure, skin thickness (mm), insulin, BMI, diabetes pedigree feature, and age), where the significance of one class of “0” is viewed as a “test negative” for diabetes and value of another class “1” is treated as “tested positive” for diabetes.

### 4.2. Performance Analysis of OWDANN Algorithm

In this section, the performance of the recommended OWDANN algorithm is analysed. Some existing methodologies are used to analyse the proposed system's efficiency, namely, “Deep Neural Network (DNN), Artificial Neural Network (ANN), Support Vector Machine (SVM), and K-Nearest Neighbour (KNN) algorithms.” In this regard, a performance comparison is carried out utilising such qualitative performance metrics, including accuracy, sensitivity, specificity, precision, recall, *f*-measure, FDR, and FPR.  Table 2 defines the device efficiency depending on the parameters above.

**Figure 4:**
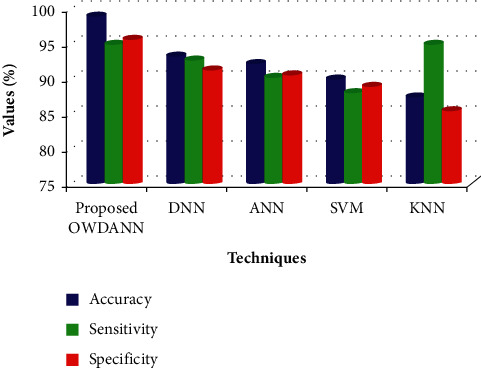
Comparative performance of the proposed OWDANN with various measures.

**Figure 5:**
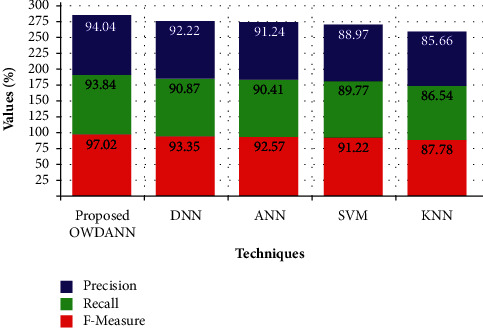
Precision-, recall-, and *f*-measure-based performance comparison.

**Figure 6:**
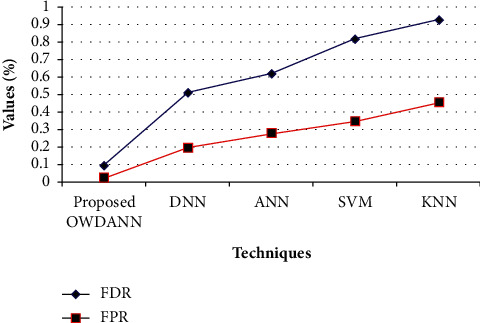
Comparative analysis of the proposed method with FDR and FPR measures.

Table 2 depicts the performance of the proposed OWDANN algorithm by comparing it with the conventional DNN, ANN, SVM, and KNN algorithm in terms of “accuracy, sensitivity, specificity, precision, recall, *f*-measure, FDR, and FPR metrics.” On analysing Table 2, the proposed one attains better performance than all the conventional DNN, ANN, SVM, and KNN algorithms. For example, the existing KNN proffers very low performance compared to the proposed one. KNN just attains 87.35% accuracy only. Also, the existing DNN, ANN, and SVM achieve lower performance than the proposed ones in terms of accuracy, sensitivity, specificity, precision, recall, *f*-measure, FDR, and FPR metrics. The proposed system attains 98.97% accuracy, 94.98% sensitivity, 95.62% specificity, 97.02% precision, 93.84% recall, 9404% *f*-measure, 0.094% FDR, and 0.023% FPR, which is greater when compared to the conventional techniques. It is more clearly explained through Figures 4–6 .

Figure 4 indicates the precision, susceptibility, and specificity metrics of the proposed OWDANN and the conventional DNN, ANN, SVM, and KNN algorithm. Precision, sensitivity, and specificity are significant metrics for disease prediction. Concerning the accuracy metric, the existing KNN algorithm proffers 87.35% accuracy only. Also, the existing SVM, ANN, and DNN approaches offer 89.94%, 92.16%, and 93.24%- accuracy, respectively, which is also lower than the proposed one. But the proposed system achieves 98.97% accuracy. Based on the sensitivity and specificity metric, the conventional KNN offers 86.46% sensitivity and 85.36% specificity. But the proposed one attains 94.98% sensitivity and 95.62% specificity, which is higher than the existing methods. Thus it concludes that the proposed OWDANN algorithm offers high-level performance than the existing methodologies.

Figure 5 shows the performance of the proposed OWDANN method with some traditional algorithms, namely, DNN, ANN, SVM, and KNN methods. Performance comparisons are made here utilising qualitative measurements like precision, recall, and the *f*-measure. The illness prediction system's performance indicators include precision, recall, and *f*-measure. From the prediction of the diabetes disease in this experiment, it is found that this proposed system has high precision, recall, and *f*-measure value. That means, the existing KNN offers precision, recall, and *f*-measure value of 87.78%, 86.54%, and 85.66%, respectively, which is very smaller than the proposed method. But the proposed method attains 97.02%, 93.84%, and 94.04%. The results show that the OWDANN technique is superior to existing methodologies in terms of precision, recall, and *f*-measure metrics for predicting diabetic illness.

Figure 6 demonstrates the performance of the proposed OWDANN technique with the existing DNN, ANN, SVM, and KNN techniques in terms of FDR and FPR. The FDR is the ratio of the number of false-positive results to the number of total positive test results. The low value of FDR and FPR improves system performance. From the figure, it can be said that the proposed OWDANN achieves 0.094% FDR, but the existing DNN, ANN, SVM, and KNN have FDR of 0.51%, 0.62%, 0.82%, and 0.93%, which is higher than the proposed OWDANN. In the consideration of FPR, the proposed technique has only 0.023% of FPR, whereas the DNN, ANN, SVM, and KNN have 0.196%, 0.279%, 0.347%, and 0.456% of FPR, respectively. Consequently, it is deduced that the proposed OWDANN has attained higher performance when contrasted to the existing system.

### 4.3. Performance Analysis of GDHC Algorithm

**Figure 7:**
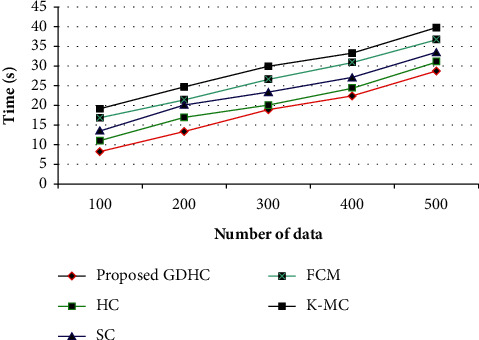
Clustering time analysis of the proposed method.

In this section, the performance of the proposed system is contrasted with the conventional Hierarchical Clustering (HC), Spectral Clustering (SC), Fuzzy C-Means (FCM), and K-Medoids Clustering (K-MC) based on clustering time. The performance is grounded on the number of data ranges from 100 to 500 data. This analysis could be graphically shown in Figure 7.

Figure 7 shows the performance of the proposed GDHC algorithm with the conventional methods, say, HC, SC, FCM, and K-MC algorithms in terms of clustering time performance metric. The time expended by the machine for operating every software is the sum of time spent. Several algorithms have passed through multiple execution cycles. Herein, the proposed one achieves higher performance than all the existing techniques. For example, for 100 data pieces, the proposed one takes 8.13 s time to cluster the data. But the existing HC, SC, FCM, and K-MC algorithms take 10.98 s, 13.45 s, 16.78 s, and 19.05 s time to cluster the data, respectively, which are higher. Similarly, for the remaining number of data pieces, the existing methodologies take more time to cluster the data when compared to the proposed one. Thus, the discussion shows that the proposed system achieves better performance than the conventional techniques.

## 5. Conclusion

Early diagnosis of the condition is the big key to determining the best cure for diabetes. In this paper, the diabetes mellitus disease prediction and severity level estimation are done by using optimal weighted based deep artificial neural network algorithm. The proposed system includes “2” phases such as disease prediction phase and severity level estimation phases. Data gathering, preprocessing, feature extraction, and classification are all the steps in the disease prediction phase. The severity level estimation phases comprise the following phases, say, preprocessing, feature extraction, and severity level prediction. The performance of the proposed system is analysed in two ways. First, the accuracy, sensitivity, specificity, precision, recall, *f*-measure, FDR, and FPR of the proposed OWDANN method are compared to those of existing DNN, ANN, SVM, and KNN algorithms. Here, the proposed one achieves 98.97% accuracy. Next, the performance of the proposed GDHC algorithm is compared with the HC, SC, FCM, and K-MC algorithms. Here again, greater efficiency was obtained than traditional approaches with the proposed scheme. In general, the findings illustrate the increased efficiency of the proposed system over current methodologies. A potential research would concentrate on changes to the model to assess all possible complications, including an organised series of possible complications. Acting with other deep learning algorithms and methods may be expanded and enhanced in future.

## Figures and Tables

**Table 1:** Association between the risk factor and diabetes.

Total score	Correlation intention
<7	Low
7–14	Moderate
15–20	High
>20	Very high

**Table 2:** Comparison of the proposed model with others.

Metrics	Proposed OWDANN	DNN	ANN	SVM	KNN
Accuracy	98.97	93.24	92.16	89.94	87.35
Sensitivity	94.98	92.67	90.21	88.01	86.46
Specificity	95.62	91.23	90.57	88.89	85.36
Precision	97.02	93.35	92.57	91.22	87.78
Recall	93.84	90.87	90.41	89.77	86.54
*F*-measure	94.04	92.22	91.24	88.97	85.66
FDR	0.094	0.51	0.62	0.82	0.93
FPR	0.023	0.196	0.279	0.347	0.456

## Data Availability

No new data were created or analysed in this research. Data sharing is not applicable to this research article.
